# Complete Genome Sequence of the Cyanobacterium *Anabaena* sp. 33047

**DOI:** 10.1128/genomeA.00809-16

**Published:** 2016-08-11

**Authors:** Sarah Pfeffer, R. Malcolm Brown

**Affiliations:** University of Texas at Austin, Molecular Biosciences, College of Natural Sciences, Austin, Texas, USA

## Abstract

This study presents the complete nucleotide sequence of *Anabaena* sp. ATCC 33047 (*Anabaena* CA), a filamentous, nitrogen-fixing marine cyanobacterium, which under salt stress conditions accumulates sucrose internally. The elucidation of the genome will contribute to the understanding of cyanobacterial diversity.

## GENOME ANNOUNCEMENT

Cyanobacteria (formerly blue-green algae) are an ancient group of diverse photosynthetic microorganisms that encompass a wide range of morphologies (1, 2). Furthermore, the extraordinary adaptive ability of these organisms has allowed them to survive in a variety of aquatic and terrestrial ecosystems. They can be found in soil environments, fresh and salt water systems, even extreme habitats such as hot springs and soda lakes (1, 2). Under saline conditions, cyanobacteria have developed mechanisms to maintain osmotic balance with the external environment through the accumulation of solutes internally (3). The genus *Anabaena*, in particular, has used this ability to survive in intertidal habitats where fluctuations between saline and freshwater environments occur frequently. The cell adapts by accumulating sucrose internally when exposed to saltwater and secreting it into the external milieu when exposed to freshwater (3). Sucrose osmolytes accumulated by *Anabaena* spp. are a potential raw material for bioethanol. Of particular interest is *Anabaena* sp. ATCC 33047 (*Anabaena* CA). It was collected in the estuarial environment of Port Aransas, Texas, USA, and exhibits a high growth rate, wide range of optimal temperature and pH, and tolerance to high irradiance (4–6). Investigations into the genome of *Anabaena* sp. 33047 may provide vital information for the understanding of this important evolved adaptation (7–9).

We have sequenced the genome of *Anabaena* sp. ATCC 33047. This particular strain has been used for a variety of studies ranging from its use in the CO_2_ removal process, outdoor cultivation for mass production of extracellular polysaccharides, study of nitrogen metabolism, and isolation and characterization of heterocysts (5, 6, 10–12). Despite these previous investigations and the importance of this strain for study, the genome of this strain had not yet been sequenced. *Anabaena* sp. ATCC 33047 was obtained from the American Type Culture Collection, and the DNA was extracted and subjected to sequencing using the Illumina HiSeq 2500 sequencer (University of Texas at Austin, ICMB Core Facility). The reads were assembled into contigs using Velvet version 1/2/02 (13) and downloaded into Geneious version 8.1.2 (14), and open reading frames (ORFs) were predicted using Glimmer (15).

The DNA sequence of *Anabaena* sp. ATCC 33047 was resolved, and bioinformatics analysis revealed that it is approximately 5.55 Mbp in size with a GC content of 41.4%. A total of 5,325 ORFs were identified in the genome, and the complete annotation of the full genome is in progress.

### Accession number(s).

This whole-genome shotgun project has been deposited at DDBJ/ENA/GenBank under the accession number LUHI00000000. The version described in this paper is the first version, LUHI01000000.
